# *Peste des petits ruminants* Virus Transmission Scaling and Husbandry Practices That Contribute to Increased Transmission Risk: An Investigation among Sheep, Goats, and Cattle in Northern Tanzania

**DOI:** 10.3390/v12090930

**Published:** 2020-08-24

**Authors:** Catherine M. Herzog, William A. de Glanville, Brian J. Willett, Isabella M. Cattadori, Vivek Kapur, Peter J. Hudson, Joram Buza, Emmanuel S. Swai, Sarah Cleaveland, Ottar N. Bjørnstad

**Affiliations:** 1 Center for Infectious Disease Dynamics, Pennsylvania State University, University Park, PA 16802, USA; imc3@psu.edu (I.M.C.); vkapur@psu.edu (V.K.); pjh18@psu.edu (P.J.H.); onb1@psu.edu (O.N.B.); 2 Institute of Biodiversity, Animal Health and Comparative Medicine, University of Glasgow, Glasgow G12 8QQ, UK; Will.deGlanville@glasgow.ac.uk (W.A.d.G.); Sarah.Cleaveland@glasgow.ac.uk (S.C.); 3 MRC-University of Glasgow Centre for Virus Research, University of Glasgow, Glasgow G61 1QH, UK; Brian.Willett@glasgow.ac.uk; 4 Nelson Mandela African Institute of Science and Technology, Arusha Box 447, Tanzania; joram.buza@nm-aist.ac.tz; 5 Department of Veterinary Services, Ministry of Livestock and Fisheries, Dodoma Box 2870, Tanzania; esswai@gmail.com

**Keywords:** epidemiology, *peste des petits ruminants*, seroepidemiologic studies, Tanzania, production system, husbandry

## Abstract

*Peste des petits ruminants* virus (PPRV) causes an infectious disease of high morbidity and mortality among sheep and goats which impacts millions of livestock keepers globally. PPRV transmission risk varies by production system, but a deeper understanding of how transmission scales in these systems and which husbandry practices impact risk is needed. To investigate transmission scaling and husbandry practice-associated risk, this study combined 395 household questionnaires with over 7115 cross-sectional serosurvey samples collected in Tanzania among agropastoral and pastoral households managing sheep, goats, or cattle (most managed all three, *n* = 284, 71.9%). Although self-reported compound-level herd size was significantly larger in pastoral than agropastoral households, the data show no evidence that household herd force of infection (FOI, per capita infection rate of susceptible hosts) increased with herd size. Seroprevalence and FOI patterns observed at the sub-village level showed significant spatial variation in FOI. Univariate analyses showed that household herd FOI was significantly higher when households reported seasonal grazing camp attendance, cattle or goat introduction to the compound, death, sale, or giving away of animals in the past 12 months, when cattle were grazed separately from sheep and goats, and when the household also managed dogs or donkeys. Multivariable analyses revealed that species, production system type, and goat or sheep introduction or seasonal grazing camp attendance, cattle or goat death or sales, or goats given away in the past 12 months significantly increased odds of seroconversion, whereas managing pigs or cattle attending seasonal grazing camps had significantly lower odds of seroconversion. Further research should investigate specific husbandry practices across production systems in other countries and in systems that include additional atypical host species to broaden understanding of PPRV transmission.

## 1. Introduction

**Figure 1:**
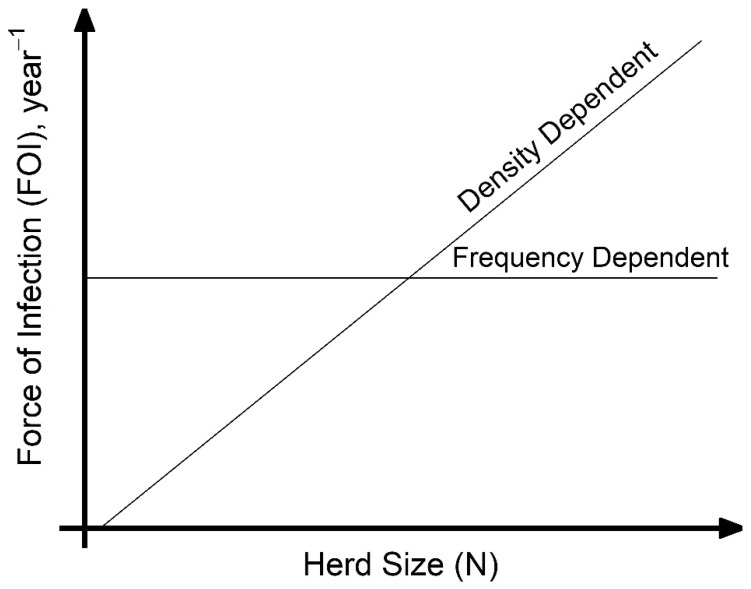
Schematic representation of the two force of infection (FOI) trajectories for two transmission pattern types with increasing household herd size (N), the count of infected hosts (I), and transmission rate β for *Peste des petits ruminants* virus at endemic equilibrium: density-dependent transmission where FOI = (β × N) × (I/N) = β × I, and frequency-dependent transmission where FOI = (β) × (I/N). Arbitrary values were used in the plot to display expected patterns for each contact rate assumption for comparison with SEEDZ data.

A key assumption made when modeling disease transmission is whether the per capita contact rate within populations is density-dependent (linearly related to population size) or frequency-dependent (independent of population size), which, respectively, results in increasing or unchanging transmission with the number of hosts. Directly estimating transmission-relevant contact rates is challenging, and empirical data on contact rate or proportion of successful transmission given contact is not commonly collected. However, calculating the force of infection (FOI), the per capita rate at which susceptible hosts become infected, from commonly collected age-specific serological studies is more tractable. Considering the case of animal diseases, under the assumption of density-dependent transmission, as the number of infected hosts increase, the FOI increases with herd size (Figure 1). Under frequency-dependent transmission, the FOI will be constant with herd size (Figure 1). Density-dependent scaling of transmission arises as the number of animals that each susceptible host interacts with increases with herd size; frequency-dependent scaling can arise if individuals form social cliques whose size is unaffected by the overall herd size. In reality, transmission scaling may be intermediate between these two extremes of scaling. When modeling for disease control, such as for the important animal pathogen *Peste des petits ruminants* virus (PPRV), the contact rate assumption chosen may have important impacts on modelling outcomes and control strategies. More empirical data is needed to inform contact rate pattern selection.

PPRV, or *small ruminant morbillivirus* (SRMV), impacts an estimated 330 million farmers worldwide across varying production systems [1]. Livestock keepers depend on sheep and goats for their livelihoods, as these animals are a source of meat, milk, and income. Household herd losses due to PPRV contribute to global poverty and food insecurity and improving control for this disease will accelerate progress on several United Nations Sustainable Development Goals, specifically goals 1 (no poverty), 2 (zero hunger), 5 (gender equality), and 10 (reduced inequalities) [2]. This socio-economically important, highly infectious virus is present in Africa, the Middle East, and Asia. It is believed that PPRV is transmitted directly via aerosols and close contact [3]. In total, PPRV currently impacts 80% of the world’s sheep and goat population [1]. The virus causes high morbidity and mortality among sheep and goats and causes an estimated $1.45–2.1 billion USD in global annual losses due to mortality, impaired production, and treatment of infected animals [1]. Furthermore, PPRV can infect other domestic hosts such as cattle and camels, as well as wild ruminants, resulting in seroconversion [3]. Due to its importance and the availability of an affordable vaccine which has demonstrated protection for up to 3 years [4], the Food and Agricultural Organization (FAO) and the World Animal Health Organization (OIE) launched a global campaign in 2015 to eradicate PPRV by 2030 [5].

Successful eradication may depend on the ability to model PPRV transmission correctly at a herd-by-herd level to inform decision making in the context of varying ecosystems, production systems and practices, herd immunity, and the presence of atypical hosts (i.e., cattle, camels, and wildlife) that may be involved in interspecific transmission [6]. PPRV transmission has been modeled previously among sheep and goats as a frequency-dependent process [7]. Interrogating this assumption with empirical data is important because if PPRV transmission follows a density-dependent process, larger herds will require disproportionally larger vaccine coverage.

Incorporating the production system into modeling efforts will also be important. The global small ruminant population is spread across diverse production systems and the population turns over rapidly, thus increasing susceptible recruitment, which increases the difficulty of implementing control strategies such as blanket mass vaccination. Previous studies have shown that pastoral production systems have significantly higher PPRV seroprevalence in Tanzania, Ethiopia, and Sudan [8,9,10,11] (Dejene 2016 unpublished MSc). However, many studies do not investigate specific husbandry practices or household herd level data for PPRV transmission. Improved investigation of specific husbandry practices will aid improved model design and parameterization in support of prioritizing efforts in the eradication campaign. To date, the majority of PPRV literature has not explored specific husbandry practices and their relationship to PPRV risk. Among the 10 published studies that do (Table 1), most were conducted in Africa. They found that specific husbandry practices were significant risk factors for increased PPRV seroprevalence, including: communal grazing [12,13,14] and watering, closed or semi-closed breeding systems [15], mixed herd species composition [16,17] and larger herd sizes [17], animal introduction to the herd [12,18,19], market exposure [17,20], and contact with other herds [16,19,20]. However, wildlife exposure was not a significant risk factor [19]. Each factor was investigated for its impact on seroprevalence in only one to three published studies, and not all multivariate analyses controlled for age. As PPR is a *Morbillivirus*, it is believed to be a fully immunizing infection that provides life-long immunity, so seroprevalence is expected to rise over age (previously demonstrated in [21]) at a rate governed by the local FOI. Thus, age-specific seroprevalence data can be used to estimate the FOI. Additionally, it would be prudent to investigate the impact of specific husbandry practices on the household herd FOI and expand knowledge of how specific practices impact PPRV transmission.

This study aims to better understand the nature and impact of contact patterns and specific husbandry practices on PPRV transmission to better inform eradication efforts. The dataset is comprised of 395 household questionnaires and accompanying cross-sectional serosurvey data of sheep, goats, and cattle managed together in households under agropastoral and pastoral production systems across 20 villages in northern Tanzania. Pastoral households were those in which livestock rearing was considered the primary livelihood activity, while agropastoral households were those in which a mix of crops and livestock were important. Given that PPRV is transmitted via aerosols and close contact, and that previous work suggests an association of larger herd size with increased PPRV seroprevalence [17], we test the hypothesis that PPRV transmission may be density-dependent at the household herd level, and therefore that the household herd FOI will increase with increasing household herd size (Figure 1). Additionally, we predict that husbandry practices that increase contact during grazing, new animal introductions, interaction with other species, or at places of aggregation (i.e., communal grazing camps) will significantly increase household herd FOI. We look at the association between these practices and the distribution of household herd FOI.

## 2. Methods

Blood samples for the cross-sectional serosurvey and household questionnaires were collected as part of the “Social, Economic, and Environmental Drivers of Zoonoses” (SEEDZ) study. The SEEDZ study data collection, data cleaning, and laboratory testing methods have been described in detail previously [11,21,26]. The relevant methods are summarized below. The SEEDZ study sampling design and questionnaire was originally designed to investigate zoonotic diseases in northern Tanzania [27,28]; however, these data are used here to study PPRV.

### 2.1. Cross-Sectional Serosurvey

Blood samples were collected in 2016 from apparent clinically healthy sheep (*Ovis aries*), goats (*Capra aegagrus hircus*), and cattle (*Bos taurus indicus*) in 20 northern Tanzanian villages in Arusha and Manyara Regions. Northern Tanzania was selected as it is a region with large livestock populations and diverse production systems. A total of 7576 animals from 417 households were sampled, and 404 household surveys were conducted using multistage random sampling (Figure S1), with details for each level of sampling previously published in [11]. Briefly, study villages were randomly selected from a spatially referenced list of 1012 villages (2012 national census, Tanzanian National Bureau of Statistics) using a generalized random-tessellation stratified sampling (GRTS) approach. This provided a spatially balanced, probability-based sample [29,30]. These households were from villages classified by expert opinion (District Veterinary or Livestock Officer) to have a primary livelihood type of either ‘pastoral’ (P) villages (those in which livestock rearing was considered the primary livelihood activity) or ‘agropastoral’ (AP) (those villages in which a mix of crops and livestock were primary livelihood activities). The GRTS procedure was stratified to select 9 villages classified as agropastoral and 11 villages classified as pastoral. Study villages were comprised of between two and four sub-villages, from which two or three were selected for inclusion in the study. The availability of animal restraint (livestock crush) was considered; otherwise, sub-village selection was random and a ‘central point’ sampling approach was used. Forty-six unique sub-villages were sampled, and the number of sub-villages per village sampled ranged from 2 to 3 (on average there are 2–3 sub-villages in agropastoral villages and 3–4 sub-villages in pastoral areas). During the sampling event, a list of all attending households was generated, and a maximum of ten households were selected using a random number generator. One household per compound (‘boma’) was sampled and up to 10 compounds per sub-village. A maximum of 10 cattle, 10 sheep, and 10 goats per selected household were randomly selected and sampled to be able to detect herd level infection (estimated at 40% for a SEEDZ target pathogen, brucellosis).

Among samples containing complete species, sex, age (by dentition), and location data, a total of 7538 serum samples were tested in duplicate using a commercially available competitive ELISA kit (Pirbright Institute, Surrey, England) directed against the hemagglutinin protein of PPRV [31]. In house specificity for this kit was estimated at 99% for all three species, and sensitivity at 88%, 81%, and 48% in sheep, goats, and cattle, respectively (Brian Willett, personal communication). Samples had been heat inactivated (56 °C, 2 h) prior to shipment to the University of Glasgow for testing. We removed 42 samples (0.6% of total) from analysis that were from households that self-reported PPRV vaccination in the past 24 months. An additional 381 samples (5.1% of total) from 20 households were removed from this analysis as they lacked a household questionnaire from which to discern self-reported PPRV vaccination. There had been no recent PPRV vaccination in the area since 2011 (vaccination history detailed further in [11]). An exploratory analysis that excluded and included these samples yielded qualitatively and quantitatively similar results with small variations (which yield slight differences in village seroprevalence and FOI by species plots in Figures S2 and S3 when compared to previous work [11] which included 7496 animals: 2080 sheep, 2419 goats, and 2997 cattle). The final serosurvey analysis sample for this study included 7115 animals (1975 sheep, 2302 goats, 2838 cattle) from 395 households. These denominators are applicable to Figures S2–S10 and Tables S1 and S2.

### 2.2. Household Questionnaires

**Figure 2:**
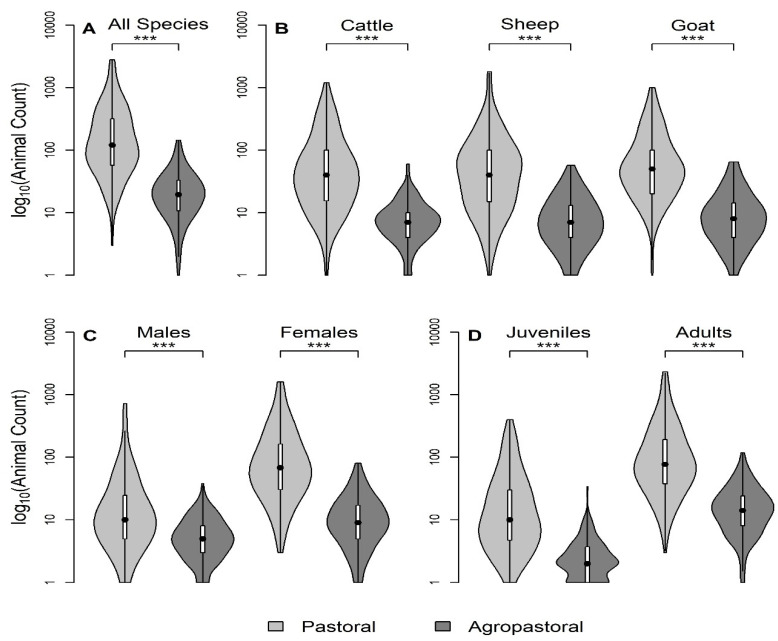
Herd size is greater in pastoral systems than agropastoral systems among livestock keeping households in northern Tanzania. Violin plots of the distribution of self-reported compound-level herd size (log_10_) by production system for the following stratifications: (**A**) all species, (**B**) by species, (**C**) all species adults by sex, and (**D**) all species by binary age groups (juvenile = no adult teeth). Herd size and stratification variables are all from household questionnaires. *** All comparisons significant (*p* < 0.001) by Kruskal–Wallis test. Boxplots are embedded within the violin (shows probability density of data at different values). All violin plots have equal maximum width.

In total, 404 household questionnaires were conducted, which included a variety of questions about the demographics and socio-economic characteristics of the household, household herd composition and demographics, and livestock health and husbandry practices. Questionnaires were piloted in Ngorongoro district in 13 households and were conducted in Swahili or local Masaai language Maa among households that were sampled during the advertised sampling event at a central location to the village. Details of variables used in this study (a subset of the full SEEDZ questionnaire) are in Supplement 2. The thirteen husbandry practice variables selected for this study were those hypothesized to increase animal contact, movement, increase the number of susceptible hosts, or allow for PPR introduction, in alignment with previous studies (Table 1). This analysis included household demographic characteristics including tribe and education level, self-reported compound herd size (sum of species specific herd sizes), seasonal grazing camp (‘ronjo’) attendance, exposure to other domestic species, and the composition of species mixtures during grazing. For the most recent 12 month period, the following variables were also investigated among all three species: introduction of new animals (yes/no), births (counts), deaths (yes/no), animal sales (yes/no), and animals given away/gifted (yes/no). Lastly, the household questionnaire contained the count of adult and juvenile animals (no adult teeth), and the count of male and female animals and these household questionnaire responses were used to stratify self-reported compound-level herd size in Figure 2.

Some variables were measured at a level different than household herd response, and these included herd size, birth counts, and counts of animal species other than cattle, sheep, and goats. These variables were all at a compound level. As previously mentioned, although a compound may be comprised of several households, only one household per compound was sampled and surveyed in the SEEDZ study. A compound was considered the relevant epidemiological unit, given that household herd animals mix in and near an individual compound and are typically held in a central enclosure overnight. In this analysis, we make the simplifying assumption that household herd was representative of the compound herd, and so the terms compound and household are used interchangeably. For the grazing variable, three responses were recorded, but the third response (cattle, sheep, goats grazed separately) only had 3 households responding, so these 3 household responses for this variable were not included in analysis when comparing the other two reported species combinations of grazing (*n* = 268).

Twelve additional questionnaire variables (species composition during confinement or watering, exposure to 10 named wildlife species) were explored for use in this study and ultimately not selected for inclusion (Supplement 2). For confinement, a majority of households responded (261/395) that cattle were kept separate from sheep and goats and there was not sufficient sample size in the other categories for comparison (all < 19 households responding). Variables relating to wildlife species exposure and watering practices only reported season (dry, wet, both) and/or had low (<50%) reporting, so these variables were not investigated further. For watering, the majority of responses were ‘both’ and for wildlife exposures most responses were ‘dry wet’ (equivalent to both), so valid statistical comparisons of FOI between different seasons was not possible, nor was presence/absence during a full year, as the households with a response all reported seeing wildlife in at least one season of the year.

An alternate classification system with smallholder as a third category, matching that of de Glanville et al. [26], was used to explore seroprevalence variation and species pair force of infection relationships at the sub-village and household scales. In that work, factor analysis for mixed data (FAMD) and hierarchical cluster analysis (HCA) were used to identify three distinct clusters which were comparable to agropastoral, pastoral, and smallholder systems. Smallholder systems generally tend to have less livestock than agropastoral systems, a small farm area (<10 hectares), and greater reliance on subsistence farming and family labor. In our sample, smallholder systems were more likely to have less livestock, grow more cash crops, have European-breed dairy cows and sell milk, and be located in areas of higher human density next to urban centers.

The final analysis dataset included 395 households from which both household questionnaire data and serosurvey data were available, and the household did not report PPRV vaccination in the past 24 months (2 households excluded). This includes a total of 7115 animals, representing 1975 sheep, 2302 goats, and 2838 cattle and these denominators apply to all analyses.

### 2.3. Analysis

To understand the role of contact pattern assumptions and husbandry practices in PPRV transmission, we calculated the force of infection (FOI, *λ*), the rate of infection of susceptible hosts, using the catalytic framework [32]. This framework provides the method for calculating the FOI from age-specific seroprevalence data from cross-sectional surveys [32,33,34,35]. According to the catalytic model, the change in age-specific seroprevalence, *P*(*a*), can be expressed as:

(1) $$\frac{dP\left(a\right)}{da}=\;\lambda \left(a\right)\left(1-P\left(a\right)\right)$$

where (1-*P*(*a*)) is the proportion of hosts still susceptible at age a, and *λ*(*a*) is the age-specific FOI. Integrating and rearranging yields:

(2) $$P\left(a\right)=1-exp\left[-\int_{0}^{a}\lambda \left(a\right)da\right]$$

which describes how the cumulative FOI up to a given age (the integrant in Equation (2)) will deplete a susceptible cohort, where *P*(*a*) represents the probability of having been infected before age *a*. Age-specific seroprevalence curves are direct empirical observations on this probability. This model assumes endemicity and that seronegatives are fully susceptible.

For this study, calculating an estimate of the household herd FOI is of interest to enable comparison by herd size, production system, and various other possible risk factors. Therefore, the simplifying assumption of a constant FOI across ages is made (previous work has shown no significant differences in FOI between age groups [21] for sheep and goats). With a constant FOI, Equation (2) simplifies to:

(3) P(a)=1−exp[−λa]. 

which upon rearrangement corresponds to the expectation of a binomial generalized linear model with a complementary log–log link:

(4) log(−log(1−P(a)))=log(ϕ)+log (a)

In Equation (4), the intercept, log(*ϕ*), represents an estimate of the log FOI when log(*a*) is used as a regression offset (i.e., has a regression coefficient fixed at unity). By using this generalized linear model, the impact of age is controlled for in this analysis of PPRV transmission heterogeneities between production systems. All sampled animals within a household herd were used to calculate the household herd FOI.

Generalized linear mixed models (GLMMs) with sex and production system type as fixed effects, log(age) as an offset, and the geographic scale (village, sub-village, household) as a random effect were used to calculate the FOI for the analysis in the main text and geographic scale analyses in supplemental material. A GLMM without production type was also explored (data not shown) but produced qualitatively similar results. The relationship between herd size and compound-level FOI was compared to expected relationships of density-dependent and frequency-dependent scaling of transmission, FOI = (β × N) × (I/N) = β × I and FOI = (β) × (I/N), respectively (Figure 1). An analysis of spatial variation in FOI and correlations in FOI between species pairs were investigated at the sub-village and household levels to determine how the patterns compared to those found at the village level in previous work [11]. Spatial autocorrelation in FOI was tested using Moran’s I Statistic. Additionally, a Local Indicators of Spatial Association (LISA) analysis was conducted to assess if there were significant spatial clusters of increased or decreased FOI at a village, sub-village, or household level [36]. A GLM with no random effect, and GLMMs with random effects at the village, sub-village, and household level were compared by Akaike information criterion (AIC) to determine which geographic scale best captured the spatial variation in FOI [37].

For calculating the household herd FOI to compare to variables from the household questionnaire (husbandry practices and births), a GLMM without fixed effects, with log(age) as an offset and household ID as a random effect was used. While the serosurvey data contained serostatus, species, age, and location information for sampled animals at various geographic scales, the household questionnaire solely contained about the entire household herd so household level GLMMs were used to calculate FOI for production system practice variables. Three single-species GLMMs and an all-species GLMM were used to calculate specific and total household (compound) herd size. Comparing the distribution of household herd FOI is more advantageous than comparing the distribution of household herd seroprevalence, as the modeling framework controls for the effects of increasing PPRV seroprevalence with age, which is expected for a fully immunizing (i.e., providing life-long immunity) infection. In our univariate analysis, differences in the distributions of household herd FOI for each husbandry practice variable response were tested with the Kruskal–Wallis test, if the assumption for heteroscedasticity was first met by the Levene’s Test. This non-parametric test was selected because husbandry practice variable response distributions did not meet the normality assumption of parametric tests. This was the preferred test through the study. If the assumption was not met, the non-parametric Wilcoxon test, and parametric oneway test and t-test as well as permutation tests (2000 permutations) were conducted to compare if the qualitative response was different between tests (significant or not).

While all aforementioned production practice variables were considered in univariate analysis to assess their relationship to the distribution of household herd FOI, a subset of these variables selected based on expert opinion were considered in multivariable logistic regression models with age group as an offset (to control for increasing seroprevalence with age) to determine their impact on PPRV seroprevalence. Variables were included based on previous knowledge from the literature (Table 1, also pig transmission [38]), aforementioned sufficient sample size in variable response levels, and findings from scaling and univariable analyses. Sheep and goat seasonal camp (ronjo) attendance were combined into a sheep or goat attendance variable due to collinearity discovered between these variables discovered using the R function vif() for GLM model variables. All analyses were conducted in R software, version 3.6.2 and 4.0.2 [39]. Generalized linear mixed models were run with the glmer function (glmer package) and the LISA analysis was conducted with the ncf package.

### 2.4. Ethics Statement

All adult participants in the SEEDZ study provided written informed consent. No data were collected from minors or children. The protocols, questionnaire, and consent procedures were approved by the following ethical review committees: Kilimanjaro Christian Medical Centre (KCMC/832, issued 27 May 2015, renewed to 16 May 2020) and the National Institute of Medical Research (NIMR/HQ/R.8a/Vol.IX/2028, approved 7 October 2015) in Tanzania; and the College of Medical, Veterinary, and Life Sciences, University of Glasgow in the United Kingdom (2001401521, approved 1 July 2015). Approval for animal work was provided by the Clinical Research Ethics Committee at the University of Glasgow School of Veterinary Medicine (39a/15, approved 15 October 2015), which authorizes research under The Veterinary Surgeons Act UK 1996 and oversees research regulated under the Animal (Scientific Procedures) Act 1986. Permission to publish this work was granted by the Director of Veterinary Services, Tanzania.

## 3. Results

### 3.1. Demographics, Herd Size, Transmission Type

Herd demographic characteristics of the 7496 serosurvey samples analyzed have been published previously (Table 1 Herzog et al. 2019) [11,21]. The characteristics of the 7115 sample subset used in this study does not differ appreciably (Table S1). Demographic characteristics of the household are found in Table S2. Most heads of household interviewed were male, the Maasai tribe comprised the largest proportion of pastoral households sampled (47.8%) followed by the Arusha tribe (11.7%), and the Iraqw tribe made up the largest proportion of the agropastoral households sampled (30.7%), followed by the Arusha tribe (17.2%). Interestingly, 96.4% and 77.8% of agropastoral and pastoral households, respectively, reported growing crops. The mean number of domestic species per household by production system type is reported in Table S3.

Most households managed all three species: sheep, goats, and cattle (*n* = 284, 71.9%). Two-species households accounted for 18.5%, with 47 households that managed cattle and goats only (11.9%), 18 that managed cattle and sheep only (4.6%), and 8 managed only sheep and goats (2%). Single-species households comprised the remaining 9.6%, with 30 households that only managed cattle, 8 that managed only goats, and none that managed only sheep. Thus, 16 households (4%) did not manage cattle, 48 (12%) did not manage goats, and 85 (22%) did not manage sheep. The highest median household herd FOI was among the sheep and goat two-species households (*n* = 8), followed by the three-species households, the rest of the two species households, and then the single-species households (Figure S7).

Pastoral (P) households had significantly larger self-reported compound herd sizes than agropastoral (AP) households, both overall and when stratified by species, sex, or age category, than agropastoral herd sizes (Figure 2A–D; Kruskal–Wallis test, *p* << 0.001). Additionally, pastoral households generally had more females than male animals (Figure 2C). Both agropastoral systems and pastoral systems typically had more adult animals than juvenile animals (Figure 2D).

**Figure 3:**
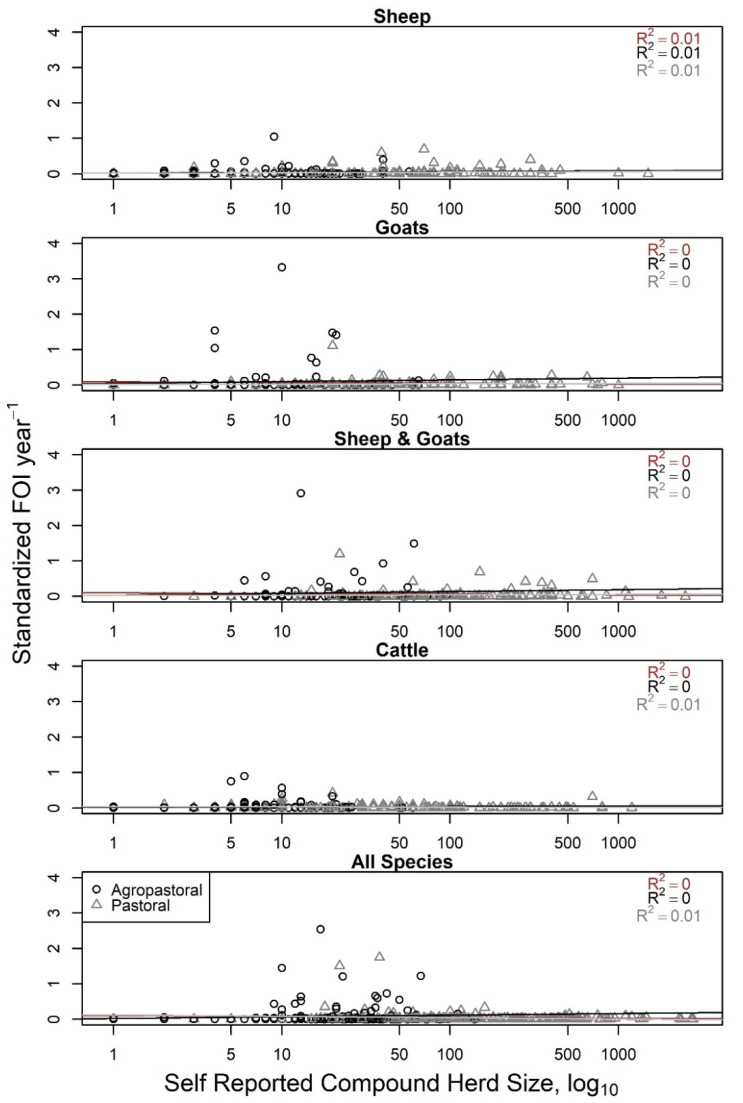
Compound herd force of infection (FOI, year^−1^) is not associated with increasing self-reported compound herd size (log_10_), consistent with overall frequency-dependent scaling of transmission patterns for *Peste des petits ruminants* virus in the compound herd. FOI values for each household/compound herd were obtained from species-specific (top four panels) or an all-species generalized linear mixed model with household as a random effect and sex and production type as fixed effects. Agropastoral compounds are represented by black, open circles; pastoral compounds by gray, open triangles. Linear models were fit to each and resulting adjusted R^2^ is reported for each production type and overall (brown). As the linear models were flat with little to no association, this matches the expected FOI pattern for frequency-dependence (constant), but not density-dependence (increasing).

Compound (household) herd FOI did not have a relationship with increasing values of self-reported compound herd size (R^2^ = 0–0.01, Figure 3), consistent with what would be expected if frequency-dependent transmission of PPRV was occurring in the compound herd among sheep, goats, and cattle (constant line in Figure 1). Household herd FOI also had a very weak relationship (R^2^ < 0.04) with compound-level birth counts in the past 12 months (Figure S8), though visually appeared to grow most rapidly when births were between 1 and 15 animals after which the relationship leveled off.

### 3.2. Patterns across Geographic Scales

Exploring variation in the PPRV seroprevalence (Figure S2) and FOI (Figure S3) across various geographic scales revealed that patterns observed at the sub-village and household herd levels were consistent with previous work [11] at the village level. Seroprevalence was higher in pastoral than in agropastoral sub-villages and households. Both production systems had more variation at finer geographic scales, and agropastoral systems had a larger number of outliers than pastoral systems. In paired species comparisons, when FOI was high for one species in a particular geographic unit, it was high for the paired species in the same unit (Figure S3), which is also consistent previous work [11] and is what would be expected if cross-species transmission were occurring among these species or if a common, external factor was affecting all three species. There was spatial variation in FOI estimates at the sub-village (Figure S4) and household herd levels (data not shown), and there was strong evidence of spatial autocorrelation in FOI for all three species at the sub-village level (Moran’s I sheep: 0.28 *p* < 0.002, goat: 0.33 *p* < 0.0001, cattle: 0. 45 *p* < 0.0001) and household level (Moran’s I sheep 0.27 *p* = 0, goat: 0.23 *p* << 0.0001, cattle: 0.23 *p* = 0). A Local Indicators of Spatial Association (LISA) analysis conducted at the village, sub-village, and household herd level (data not shown) indicated significant spatial clusters of increased and decreased FOI only at the sub-village scale (Local Moran’s I, *p* < 0.05; Figure S5). The lowest AIC among GLMMs with no random effect, and random effects at the village, sub-village, and household level was for the model with sub-village as a random effect (Table S4). The addition of the smallholder classification to this study’s dataset based on [26] did not enhance predictability of the variation patterns in FOI or PPR seroprevalence seen at finer geographic scales (Figures S9 and S10).

### 3.3. Specific Husbandry Practices and PPRV Risk

**Figure 4:**
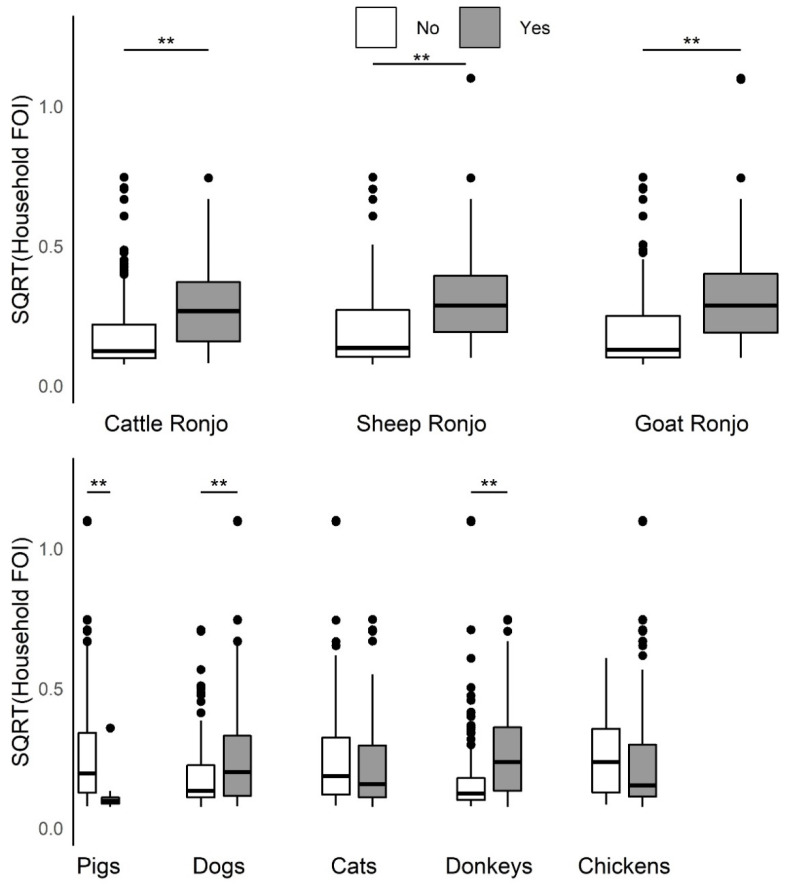
Boxplots of the distribution of the household herd force of infection (FOI, square root transformed, unit = year^−1^) by seasonal grazing camp attendance for each species in the past 12 months and presence/absence of additional domestic species managed at the household. There was a significant increase (Kruskal–Wallis test, *p*-value < 0.005) in the household herd FOI for households that reported cattle, sheep, or goats going to a seasonal grazing camp (‘ronjo’) in the past 12 months as well as households that reported having dogs or donkeys. Domestic pigs were associated with a significant decrease in household herd FOI. ** Comparison significant (*p* < 0.005) by Kruskal–Wallis test.

**Figure 5:**
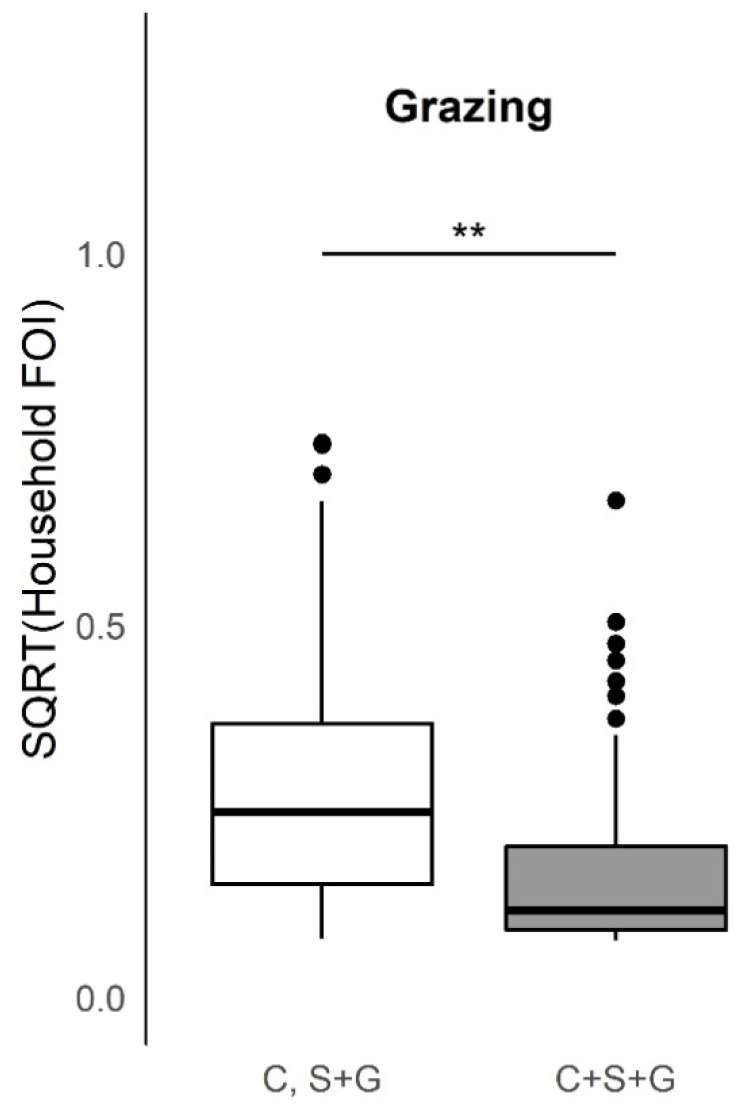
Boxplots of the distribution of the household herd force of infection (FOI, square root transformed, unit = year^−1^) by varying species composition during grazing practices. There was a significant increase (Kruskal–Wallis test, *p*-value < 0.005) in the household herd FOI for households that reported grazing cattle (C) separately from combined sheep and goats (S+G) as opposed to all three species together (C+S+G). ** Comparison significant (*p* < 0.005) by Kruskal–Wallis test.

Household herd FOI increased significantly (Kruskal–Wallis test, *p*-value < 0.005) among households that reported any species in their herd attended a seasonal grazing camp (Figure 4) or when the household also managed dogs or donkeys (Figure 4). Additionally, household herd FOI increased significantly when cattle were grazed or confined separately from sheep and goats as opposed to all three species together (Figure 5), or when cattle or goats were introduced to the compound, animals died in the herd, or herd animals were sold, or given away in the past 12 months (Figure S6). Household herd FOI significantly decreased when the household also managed pigs (Figure 4), however; only agropastoral households reported this practice. Household herd FOI was not significantly higher or lower when sheep were introduced to the compound (Figure S6), or when households also managed cats or chickens (Figure 5). Additionally, FOI was not significantly higher or lower when household herd livestock were grazed with animals from another compound (data not shown, ‘yes’ reported by 40% of the 372 households that answered the question). Ninety-five households reported that 2 or more households were present in the same compound (24% of household questionnaires; 6 agropastoral and 89 pastoral, or 3% and 44%). Among these, the FOI was not significantly higher or lower when animals were grazed with other livestock in the same compound (data not shown, ‘yes’ reported by 84% of the 73 households that answered the question). Although some households used more than one grazing practice, grazing was predominantly conducted by herding animals (range of households responding for all three species: 74.2–89.9%). Purchased livestock mainly came from the market (cattle: 78.5%, sheep: 82.6%, goats: 73.4%), and livestock were sold mostly at market (cattle: 80%, sheep: 73.4%, goats: 83.2%).

The majority of households stated that they did not keep livestock elsewhere (72.2% overall, 77.1% AP, 74.3% P). Among households who did, 72%, 31%, and 30% reported cattle, sheep, and goats, respectively, being kept elsewhere, with grazing as the top reason for all three species. The mean number kept elsewhere was 18.4 cattle, 17.1 goats, 25.9 sheep among agropastoral households and 74.4 cattle, 43.5 goats, and 76.1 sheep among pastoral households. Owners also kept animals they did not own (13.6% reported keeping cattle, 2.5% sheep, and 3.8% goats) with the top three reasons being a favor, milk loan (cattle) or breeding loan (sheep, goats), or grazing. For households that reported introducing animals, the most common practice was to mix new animals into the herd without a period of quarantine (47.7% cattle and 65.3% sheep and goats), or both mix and provide medicine to new animals (6.3% cattle and 10.6% sheep and goats). Sample sizes for categories other than mixing were not sufficiently large to conduct statistical testing (all *n* ≤ 11).

Multivariable logistic regression (Table 2) revealed that species, production system type, and goat or sheep introduction or seasonal grazing camp attendance, cattle or goat death or sales, or goats given away in the past 12 months significantly increased odds of seroconversion, whereas managing pigs or cattle attending seasonal grazing camps had significantly lower odds of seroconversion. Total herd size, dogs, and donkeys were not included in this model based on lack of relationship between compound herd size and FOI, and on the expert opinion that involvement in PPRV transmission was not likely relevant (or biologically plausible).

## 4. Discussion

This study provides evidence of important heterogeneities in PPRV risk at the household herd level. Overall, the absence of an increase in FOI with herd size, as would be predicted if transmission scales in a density-dependent fashion, suggests that contact rates are socially clustered within the household herds and may follow a frequency-dependent scaling of transmission. Specific husbandry practices associated with significantly higher household herd FOI in univariable analyses included attendance at seasonal grazing camps (‘ronjos’), cattle or goat introduction to the compound, grazing cattle separately from sheep and goats vs. all together, presence of donkeys or dogs kept by the household, the report of any animal deaths, sales, or gifting (given away) in past 12 months. Multivariable analyses found goat or sheep introduction or seasonal grazing camp attendance, cattle or goat death or sales, and goats given away in the past twelve months to be significant production practices that increased odds of PPRV seroconversion, whereas managing pigs, and cattle seasonal grazing camp attendance decreased the odds of seroconversion. Additionally, spatial patterns in PPRV seroprevalence and FOI as well as paired species FOI comparisons observed at the sub-village and household herd level were consistent with previous village-level work [11].

This study quantified and used herd size variation among households in northern Tanzania to explore hypotheses about contact rate assumptions on PPRV transmission. The findings are interesting in that evidence of frequency-dependent scaling of transmission was present down to a finer scale than expected: the compound (household) scale. This pattern was consistent for both agropastoral and pastoral systems. The geographic scale at which transmission is assessed is important—within a herd, one might expect to see density-dependent transmission as larger numbers of animals may be herded more closely, but at a larger geographic scale, one may expect to see frequency-dependent scaling of transmission as animals are managed in discrete herd units. In fact, both the mode of transmission and the underlying contact network structure drive patterns seen at different scales (within and between populations) and have been previously shown to give paradoxical predictions in other *Morbillivirus* systems such as human Measles virus [40]. Future research to develop within-household and within-compound herd contact networks under different husbandry practices to tease apart mixing would provide further key insight as to when this pattern may break down. Furthermore, for future dynamical modeling, it would be prudent to explore transmission mixing patterns intermediate between density-dependent and frequency-dependent transmission, as evidence from elk and vole wildlife systems has supported modes of transmission between classically defined density- and frequency-dependent modes [41,42].

In a further refinement of the distinction between pastoral and agropastoral households regarding growing crops, de Glanville et al. [26] used multiple factor and hierarchical cluster analysis to classify Tanzanian households into three clusters that broadly mapped onto conventional definitions of agropastoral, pastoral, and smallholder systems. There have been many proposed approaches to defining production systems [43], but these approaches have been changing over time (reviewed in [26]). This study identifies possible evidence that agropastoral systems may be too broad of a definition at a household level (Figure S2) and possibly sub-village level as there is a larger number of outliers in PPRV seroprevalence, suggesting that some other household characteristic or husbandry practice is missing from traditional definitions. Given the difficulties in production system classification, it may be important to keep studying specific husbandry practices in more detail, in order to prioritize vaccination to areas where these practices are prevalent (or times when they are prevalent) and to encourage changes to the practices with the highest transmission risk. Such targeted vaccination and altered practices are likely to complement ongoing PPRV vaccination control efforts towards the goal of PPRV eradication. The addition of the smallholder classification to this study’s dataset based on [26] did not appreciatively enhance predictability of the variation patterns in FOI or PPR seroprevalence seen at finer geographic scales (Figures S9 and S10) but, in this dataset, not many households were considered ‘smallholders’ and therefore purposeful sampling in a smallholder production system may be useful in future studies.

This study explored husbandry practices for cattle managed alongside sheep and goats and their relationship to PPRV transmission risk. The univariable finding that cattle introduction to the compound increases household herd FOI may result from cattle introductions occurring at the same time as sheep or goat introductions (which may be PPRV infected and shedding virus). This finding is counter balanced by the univariable evidence presented in this study that grazing cattle with sheep and goats was associated with a significantly lower household herd FOI compared to when cattle are separated, and is consistent with the prevailing dogma and experimental transmission findings [44,45] that cattle are likely dead-end PPRV hosts. However, the exact role of cattle in transmitting PPRV is still under experimental investigation [44,45]. Multivariable analysis revealed that goat and sheep, but not cattle, introduction was a significant production practice associated with increased PPRV seroconversion. Future longitudinal research in multispecies herds to document species composition and timing of livestock introductions within different production systems will better inform prioritization of interventions towards PPRV eradication. The univariable finding that PPRV household herd FOI was higher with the presence of dogs or donkeys may need to be investigated further (while both are significant when added to the multivariable model of Table 2, data not shown because not believed to be major contributors to PPRV transmission). In this dataset, more pastoral households reported having donkeys than agropastoral households, which could explain the pattern seen, whereas dogs were reported approximately equally across the production system types. Having donkeys or dogs may be associated with another production system practice not investigated in this study that could influence PPRV transmission. This study supports the importance of conducting more PPRV research on the role of ‘atypical’ species ahead of the global eradication campaign, as called for by members of the PPR Global Research and Expertise Network (GREN) [46].

Overall, the husbandry practices that increase PPR transmission risk that were identified in this study align with and expand upon previously published studies (Table 1). Like these studies, husbandry system, grazing system (specific combinations of species), herd species composition, and animal introductions (compound level) were associated with a significant increase in household herd PPRV transmission risk. PPRV risk was higher in pastoral systems where herd sizes were significantly larger and where PPRV is likely to be endemic.

This study made several assumptions, consistent with previous work in this population [11,21], including the following: PPRV endemicity in Tanzania; a positive cELISA result indicates past PPRV exposure and current protection; a negative cELISA result indicates no past exposure and current susceptibility. Furthermore, this analysis assumed that the BDSL cELISA kit was suitable for testing cattle samples and that cross-reactivity with rinderpest or rinderpest-like viruses [47] was not expected in these samples given the sampling date and methods used to develop the cELISA kit. These assumptions and the modeling assumptions made in this analysis have previously been discussed [11,21] as reasonable first approximations for understanding PPRV transmission dynamics. Based on our findings in age seroprevalence curves in [21], the assumption of endemicity at a household level is likely to be reasonable for pastoral households, but may be less appropriate for agropastoral households. Additionally, the role of cattle in PPRV transmission, which is an active area of research [38,44,45], has similarly been discussed [11,21]. While this investigation of specific husbandry practices aligned with previously published findings, this work provides a more complete investigation of the distribution of herd size across compounds, two production systems, and three species and addresses their relevance to the FOI. Indeed, most of these previously published findings come from a small handful of African or Middle Eastern countries and it would be beneficial to compare to husbandry practices across a wider geographic scale. Finally, compound-level herd size (reported as species specific and summed to all species) was self-reported based on the randomly selected household from that compound. Therefore, herd size counts may be subject to rounding, recall bias, or culturally specific response bias due to sensitivity in answering this question. However, the questionnaire method for recording herd size is often a more tractable approach in the field, especially among pastoralists whose herds are impractical to count due to size or distribution in several locations. Although there was no reluctance to answer this question in our study, respondents may have underreported herd size, for example if concerned about taxation. If counts were systematically underreported, then we do not expect an impact on our results qualitatively. However, self-reported compound-level herd size should be considered approximate.

This study explored evidence for density- or frequency-dependent transmission scaling of PPRV and husbandry practices that impact PPRV transmission risk among households in northern Tanzania. Taken together, the data presented here support the use of frequency-dependent transmission scaling in PPRV modelling work at finer geographic scales and identified specific husbandry practices associated with increased household herd transmission risk. This analysis also quantified the distribution of herd sizes in pastoral and agropastoral production systems in the study region in total and by species, sex, and age group stratifications. Future research should assess model sensitivity to variations in contact rate assumptions, and the resulting impact on proposed PPR control strategies. Additionally, future studies should explore the relationship between FOI and herd size in settings where both household-level and compound-level herd sizes can be confirmed by counting animals instead of self-reporting in order to reduce potential bias. This study found that specific husbandry practices such as seasonal grazing camp (‘ronjo’) attendance and livestock introduction practices are associated with increased household herd PPRV transmission risk. Given that an affordable vaccine is available, vaccination should be the cornerstone of control efforts to reduce transmission at camps and during new animal introductions when available. Furthermore, if additional husbandry practices are found to be associated with increased PPRV transmission risk in future research, then vaccination could be targeted to settings in which these practices are common. In settings lacking access to veterinary services and vaccination, further research may be needed to determine which alterations to husbandry practices reduce transmission risk, such as modifications at seasonal grazing camps without restricting attendance and standardizing best practices for animal introductions to reduce PPRV transmission.

## Figures and Tables

**Table 1:** Husbandry and PPRV Transmission Risk from Multivariable Studies.

Practice	Significance in PPRV Transmission	Location	Reference	SEEDZ Study Variable *
**Overall Husbandry System**	Significant, pastoral higher	Ethiopia, Sudan, Tanzania	Dejene (2016, unpub. MSc), Salih et al. 2014 [10], Kivaria et al. 2013 [8], Swai et al. 2009 [9], Herzog et al. 2019 [11], Herzog et al. 2020 [21]	production system type
**Within Herd**				
*Animal housing type*	Significant	Sudan	Salih et al. 2014 [10]	confine_csg
*Breeding system*	Significant, highest in closed or semi-closed vs. open	Libya	Almeshay et al. 2017 [15]	
*Herd size*	Significant, larger herd at increased risk	Jordan, Tanzania	Al-Majali et al. 2008 [17]	cattle/goats/sheep number
*Grazing system*	Significant in Tanzania (communal), Democratic Republic of the Congo (DRC, communal, free-ranging); significant in sedentary highland systems in Ethiopia	Tanzania, DRC, Ethiopia	Mbyuzi et al. 2014 [12], Nkangaga (2014, unpub. MSc) Bwihangane et al. 2016 [22], Agga et al. 2019 [14]	production system type, cattle/goats/sheep ronjo,, freerange, herded, tethered, zero
*Mixed herd composition*	Significant, in Jordan only for sheep, not goats	Algeria, Jordan	Kardjadj et al. 2015 [16], Al-Majali et al. 2008 [17]	cattle/goats/sheep number
*Water source*	Significant, shared water sources higher risk than on-farm sources	Ethiopia	Dejene (2016, unpub. MSc)	goats_water_samecattle, sheep_water_samegoats, sheep_water_samecattle
*Wildlife exposure*	Not significant	Tanzania	Torsson et al. 2017 [19], Chota et al. 2019 [20]	see_buffalo, antelope, wildebeest, wildpigs
**Movement**				
*Introduction of new animals*	Significant in most studies; not significant for one of the two Tanzanian studies.	Sudan, Tanzania	Saeed et al. 2018 [18], Mbyuzi et al. 2014 [12], Nkangaga (2014, unpub. MSc), Torsson et al. 2017 [19]	cattle/goats/sheep_intro cattle/goats/sheep_born
*Origin of animal*	Significant, in Libya: highest in imported animals, followed by animals from market	Ethiopia, Libya	Dejene (2016, unpub. MSc), Almeshay et al. 2017 [15]	
*Contact with other herds*	Significant; not significant for other domestic herds (Tanzania)	Algeria, Tanzania	Kardjadj et al. 2015 [16], Torsson et al. 2017 [19], Chota et al. 2019 [20]	
*Contact at live animal markets*	Significant	Jordan	Al-Majali et al. 2008 [17], Chota et al. 2019 [20]	
*Migration*	Significant	India	Mahajan et al. 2012 [23]	
**Services**				
*Absence of veterinary services*	Significant	Jordan	Al-Majali et al. 2008 [17]	vaccine, vaccine_which, vaccine_other
*Mixing sick and healthy animals*	Significant, this practice due to labor shortage	Kenya	Kihu 2012 [24], Kihu et al. 2013 [25]	quarantine_cattle, quarantine_shoats

* Variable dictionary available in Supplement 2.

**Table 2:** Multivariable logistic regression results assessing the relationship between specific production system variables and PPRV seroconversion.

Variable	Odds Ratio (*e*^β^)	95% Confidence Interval	*p*-Value
Species—Sheep	4.05	(3.27, 5.03)	*p* << 0.001
Species—Goat	4.17	(3.39, 5.14)	*p* << 0.001
Pastoral production	4.63	(3.47, 6.21)	*p* << 0.001
Managed pigs (yes)	0.01	(0, 0.04)	*p* << 0.001
Cattle introduction (yes)	1.07	(0.89, 1.29)	*p* > 0.1
Goat introduction (yes)	1.48	(1.22, 1.80)	*p* << 0.001
Sheep introduction (yes)	0.68	(0.54, 0.86)	*p* < 0.01
Cattle death (yes)	1.47	(1.2, 1.79)	*p* << 0.001
Goat death (yes)	1.38	(1.1, 1.73)	*p* < 0.01
Sheep death (yes)	1.05	(0.85, 1.3)	*p* > 0.1
Cattle sold (yes)	1.35	(1.1, 1.65)	*p* < 0.01
Goat sold (yes)	0.67	(0.54, 0.83)	*p* << 0.001
Sheep sold (yes)	0.98	(0.80, 1.19)	*p* > 0.1
Cattle given away (yes)	1.19	(0.96, 1.48)	*p* > 0.1
Goat given away (yes)	1.39	(1.12, 1.74)	*p* < 0.01
Sheep given away (yes)	0.89	(0.69, 1.14)	*p* > 0.1
Cattle ronjo seasonal camp attendance (yes)	0.64	(0.48, 0.83)	*p* < 0.01
Sheep or goat ronjo seasonal camp attendance (yes)	1.57	(1.24, 2.0)	*p* << 0.001

Reference groups: species—cattle, production system—agropastoral, grazing—cattle, sheep, goats all separated, and all other variables—no.

## Data Availability

The anonymized dataset used in this study will be made available on the following repository: http://researchdata.gla.ac.uk/.

## Supplementary Materials

The following are available online at https://www.mdpi.com/1999-4915/12/9/930/s1, Figure S1. SEEDZ cross-sectional serosurvey and household questionnaire study sampling design and geographic scales; Figure S2. Boxplots of variation in PPRV seroprevalence by species and production system at the village level, sub-village level, and household herd level; Figure S3. Standardized force of infection (FOI, year^−1^) estimates at the village level (previous work [19]), sub-village level, and household herd level by species; Figure S4. Spatial variation in the standardized force of infection (FOI, year^−1^) estimates; Figure S5. Spatial variation in the mean force of infection (FOI, year^−1^) estimates at the village and sub-village level using Local Indicators of Spatial Association (LISA); Figure S6. Boxplots of the distribution of the household herd force of infection (FOI, square root transformed, unit = year^−1^) by various husbandry practices in the past 12 months; Figure S7. Boxplots of the distribution of the household herd force of infection (FOI, square root transformed, unit = year^−1^) among households managing one, two, and three species; Figure S8. Household herd FOI had a very weak relationship with compound-level birth counts in the past 12 months; Figure S9 Boxplots of variation in PPRV seroprevalence by species and production system at the sub-village level and household herd level; Figure S10. Standardized force of infection (FOI, year^−1^) estimates by cluster (pastoral, agropastoral, smallholder based on de Glanville et al. 2020 [13] classifications) at the sub-village and household herd level by species; Table S1. Sample population characteristics; Table S2. Demographic characteristics of the 395 surveyed heads of household; Table S3. Household-level domestic species composition by production type; Table S4. Generalized linear model (GLM) and generalized linear mixed model (GLMM) comparison shows that the sub-village geographic scale best captures spatial variation in *peste des petits ruminants* virus (PPRV) transmission risk.
