# Supplementary material for: Peste des petits ruminants Virus Transmission Scaling and Husbandry Practices That Contribute to Increased Transmission Risk: An Investigation among Sheep, Goats, and Cattle in Northern Tanzania
Source: Viruses. 2020 Aug 24;12(9):930. doi: 10.3390/v12090930 (PMC7552010; doi:10.3390/v12090930)
Supplement: Supplementary file 1 [file viruses-12-00930-s001.zip › supplementary file.pdf]

viruses

MDPI

Supplemental Information

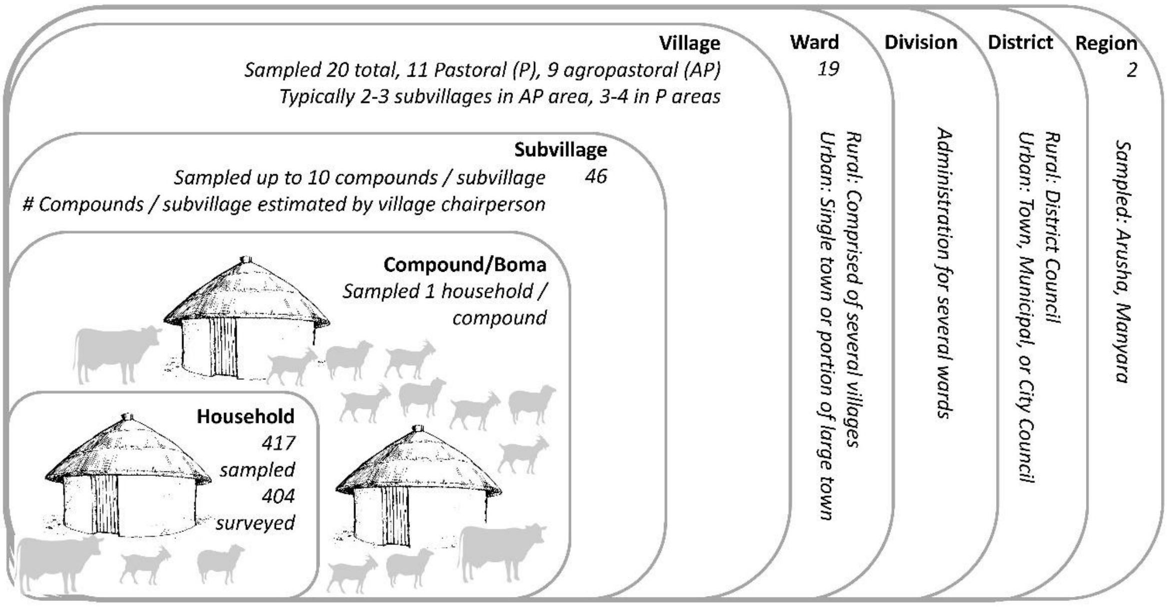

Figure S1. SEEDZ cross-sectional serosurvey and household questionnaire study sampling design and geographic scales.

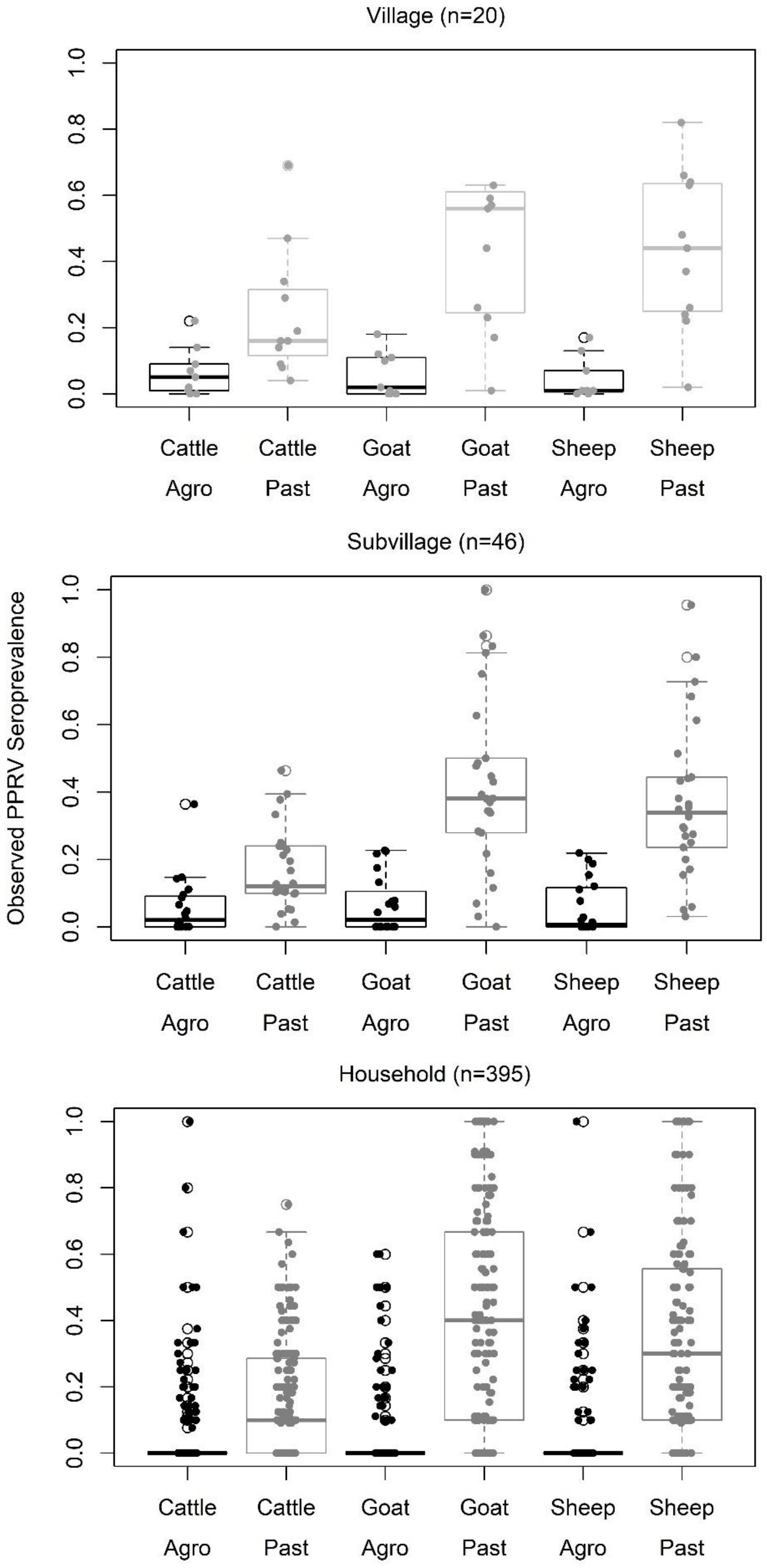

Figure S2. Boxplots of variation in PPRV seroprevalence by species and production system at the village-level, sub-village level, and household herd level. Jittered dots are seroprevalence proportion estimates of sampled villages, sub-villages, and household herds, respectively. Agro = Agropastoral (Black), Past = Pastoral (Grey).

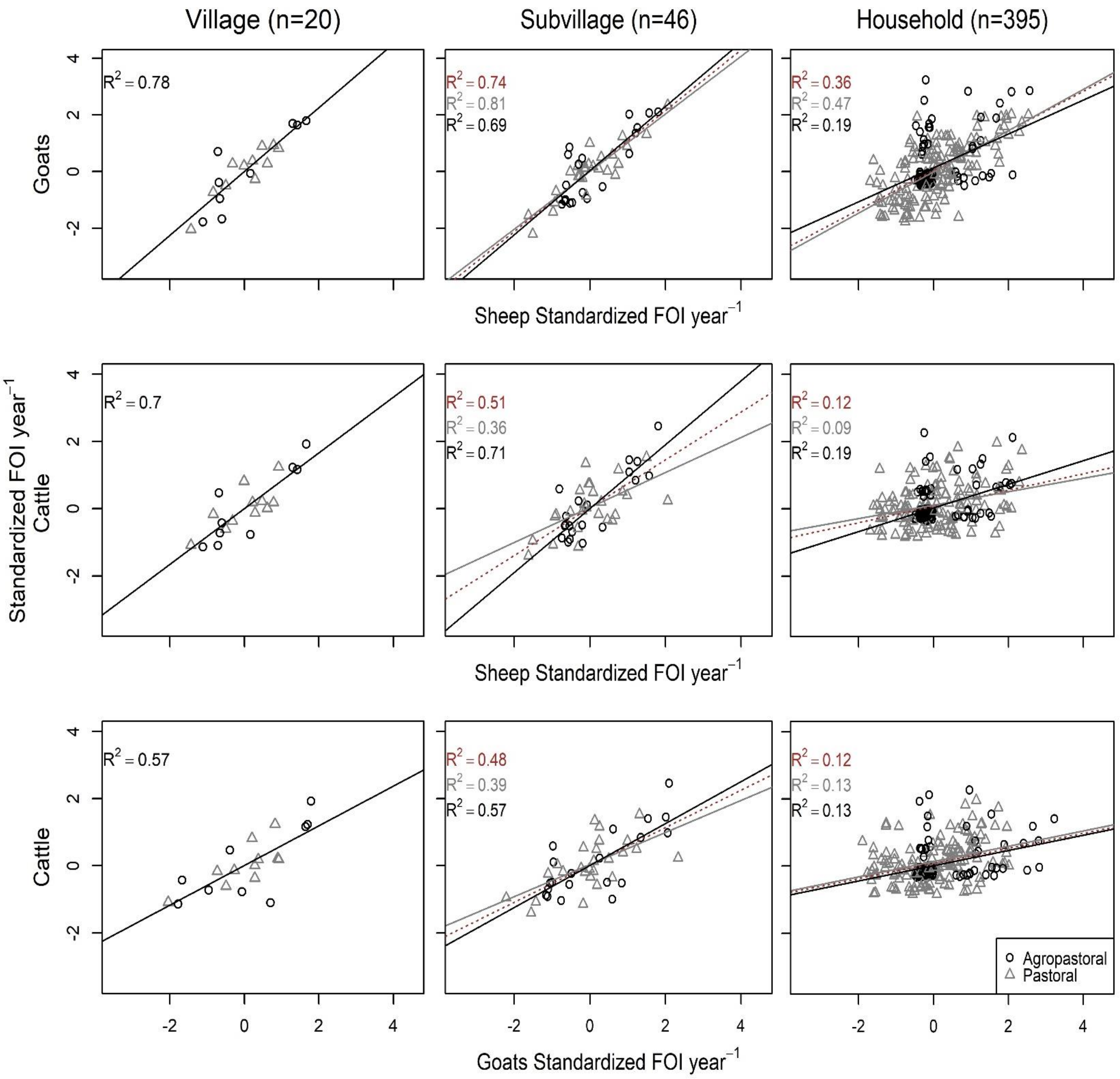

Figure 3. Standardized force of infection (FOI, year^{-1}) estimates at the village-level, sub- village. **level,** and household herd level by species. Mean FOI is standardized to zero and is in the middle of both axes. Where the FOI was higher or lower for one species, it was also higher or lower than the mean for the second species plotted. These patterns, at all scales, are consistent with what would be expected for cross-species transmission or if a common, external factor was affecting all three species. All correlations were are significant (most $p < 0.001$; all $p < 0.002$). Linear models were fit to each production type and resulting adjusted R^2 is reported for each production type and overall (brown).

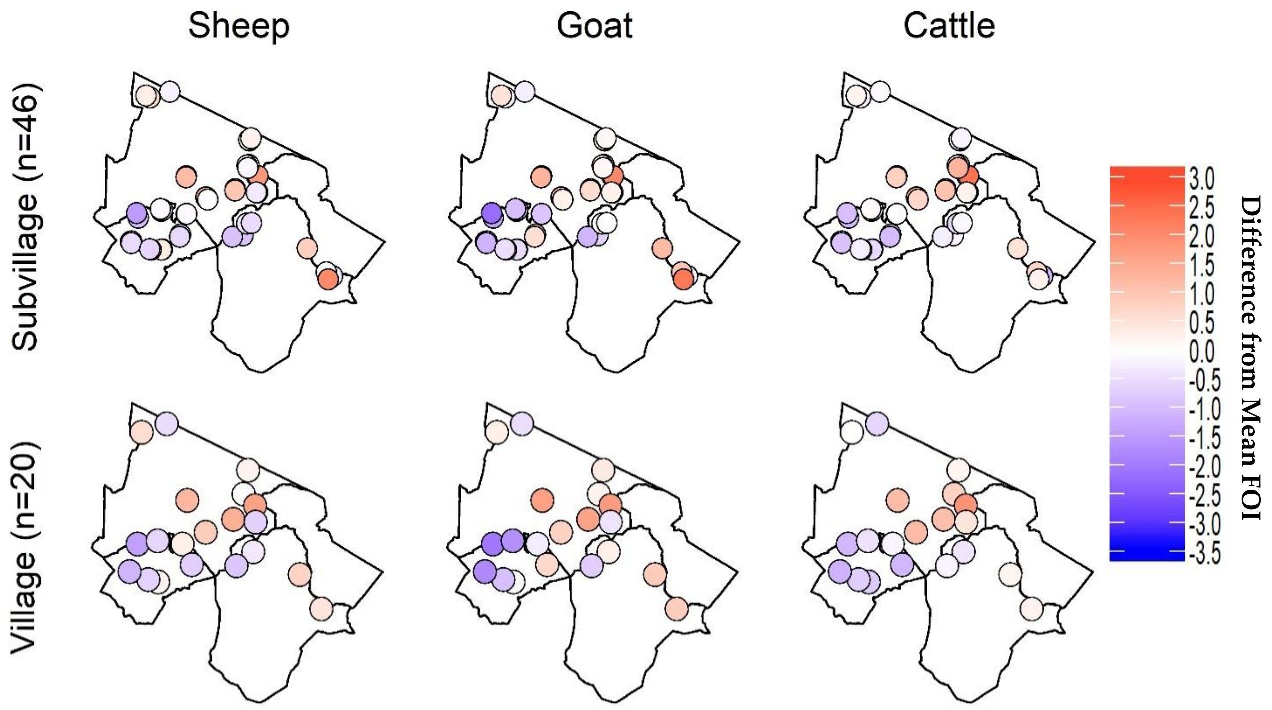

Figure 4. Spatial variation in the standardized force of infection (FOI, year^{-1}) estimates at the village and sub-village level based on FOI calculated from a generalized linear mixed model controlling for sex and production system (expert opinion) with geographic scale as the random effect.. Map is of northern Tanzania study region (see Herzog et al 2019, main text reference 20, Figure 1). Household herd analysis run (395 households), but map data not shown here for identity protection. Blue coloring indicates a FOI that was lower than the mean FOI (standardized to zero) and red coloring indicates a FOI that was higher than the standardized mean FOI. At the village-level, there was strong evidence of spatial autocorrelation (Moran's I) in FOI for goats and cattle $p < 0.001$) but weak evidence in sheep (0.28, p= 0.08). There was strong evidence for all three species at the sub-village level (sheep: 0.28 $p < 0.002$, goat: 0.33 $p < 0.0001$, cattle: 0. 45 $p < 0.0001$) and household level (sheep 0.27 $p = 0$, goat: 0.23 $p << 0.0001$, cattle: 0.23 $p = 0$) .

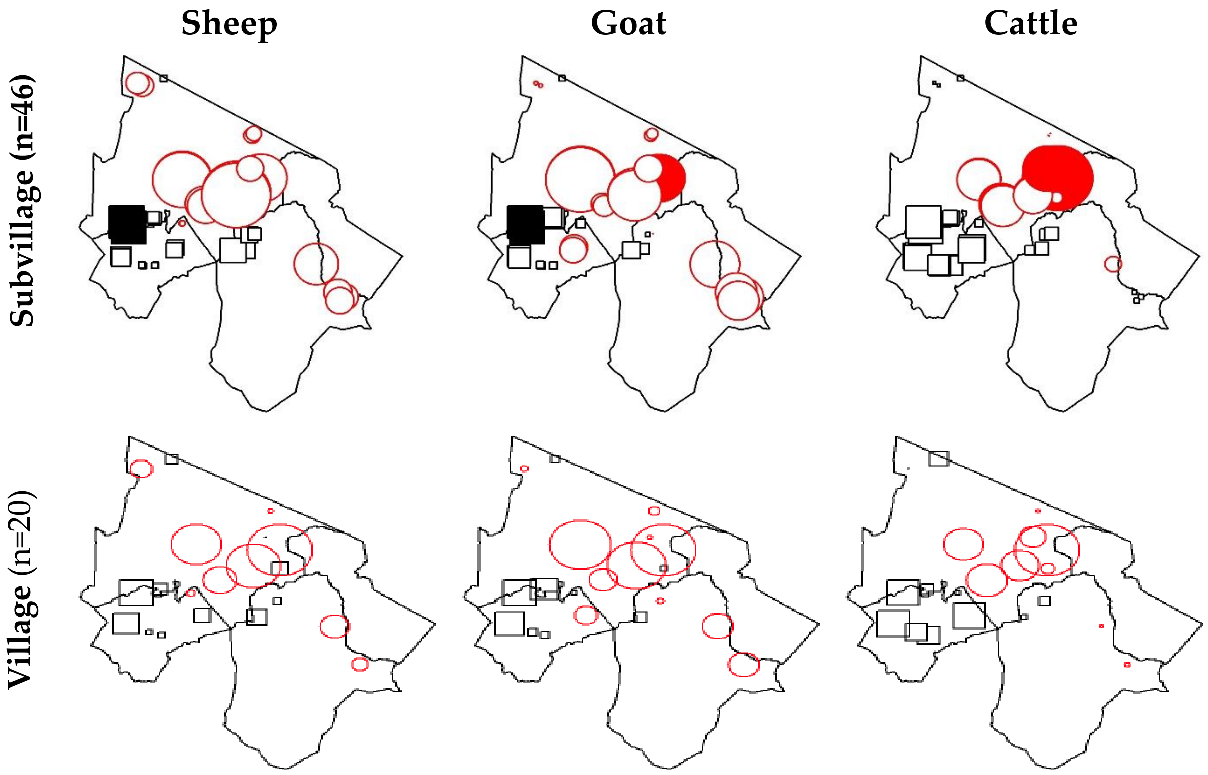

Figure 5. Spatial variation in the force of infection (FOI, year^{-1}) estimates at the village and sub-village level using Local Indicators of Spatial Association (LISA) based on FOI calculated from a generalized linear mixed model controlling for sex and production system (expert opinion) with geographic scale as the random effect. Household herd analysis run (395 households), but data not shown here. Spatial clusters of increased FOI are in red, and spatial clusters of decreased FOI are in black. Filled in shapes indicate significant spatial clusters of increased or decreased FOI based on a permutation test (500 re-samples) two-sided p-value at the 5% level. No significant clusters were seen at the village or household level (household data not shown).

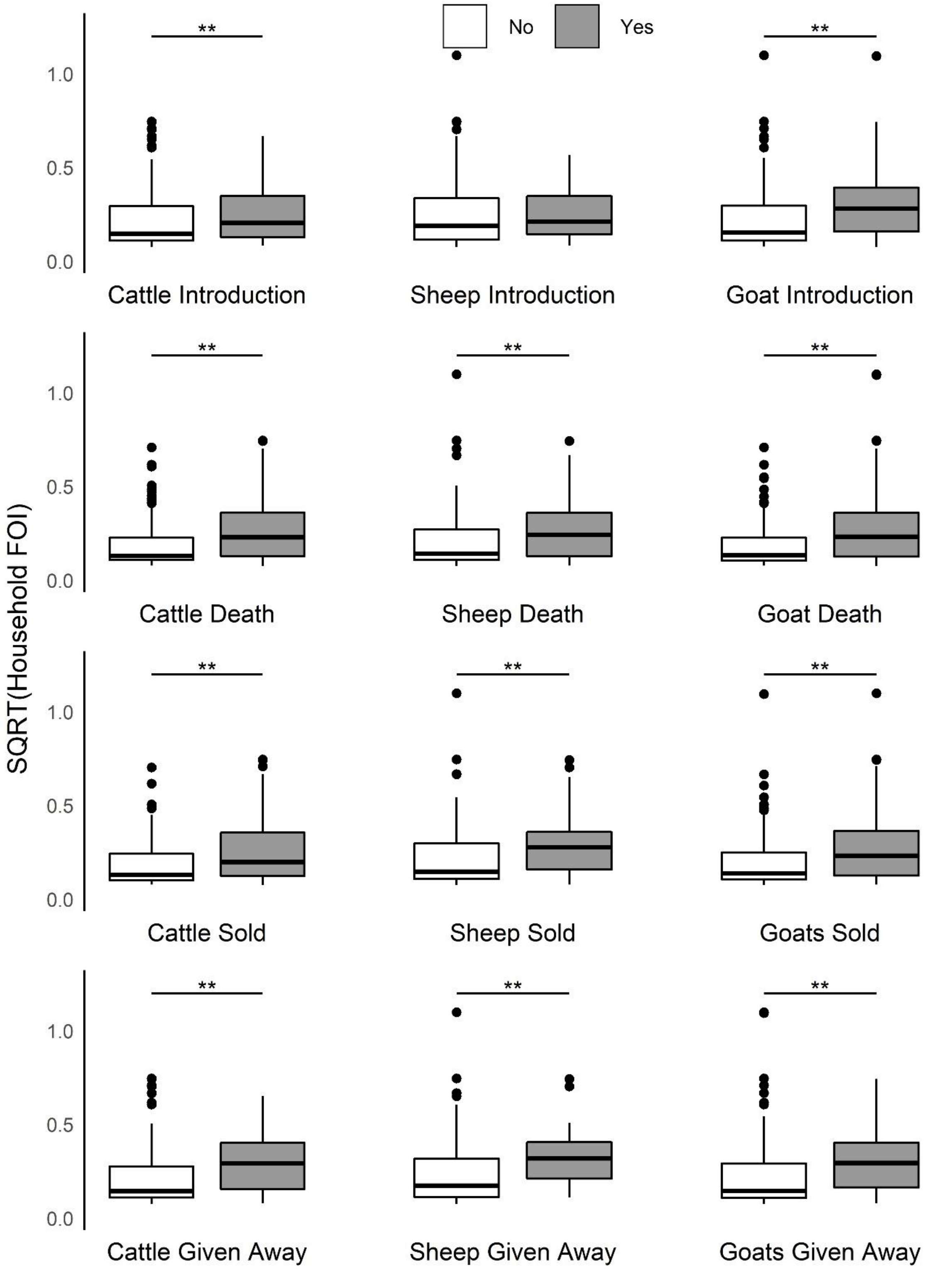

Figure 6. Boxplots of the distribution of the household herd force of infection (FOI, square root transformed, unit = year^{-1}) by various husbandry practices in the past 12 months. There was a significant increase (** Kruskal-Wallis test, p-value < 0.005) in the household herd FOI for households that reported cattle or goat introductions as well as death, sale, or giving away (gifting) any species in the past 12 months.

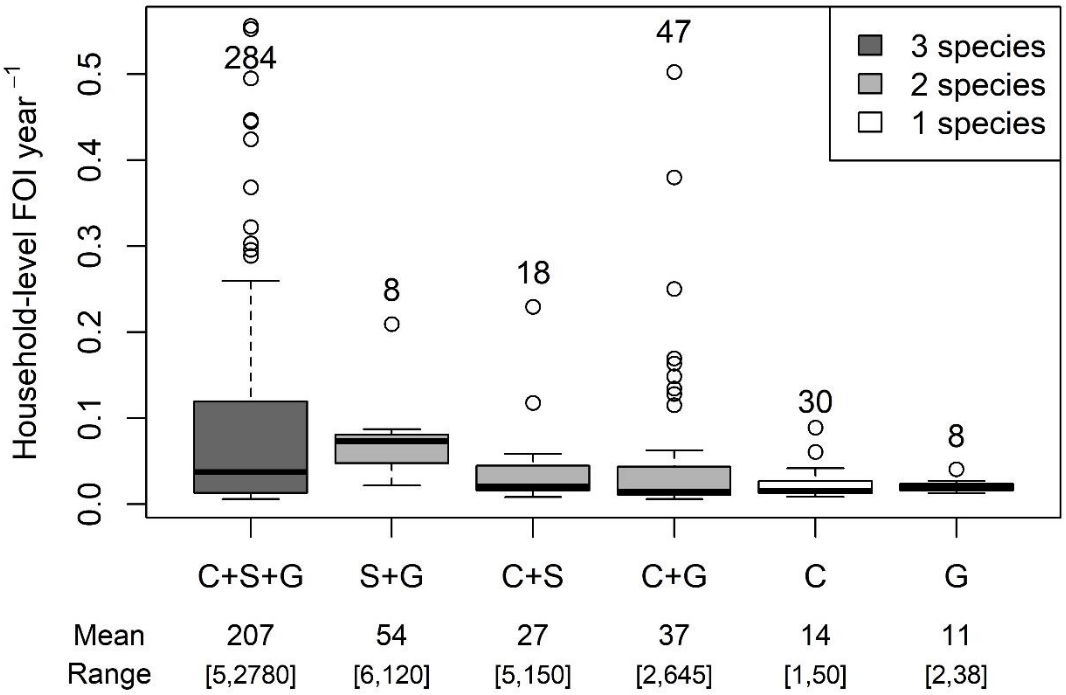

Figure 7. Boxplots of the distribution of the household herd force of infection (FOI, square root transformed, unit = year^{-1}) among households managing one, two, and three species. C= cattle, S = sheep, G = goats. Number of households in each category are annotated inside the plot and the mean and range of household herd size are annotated beneath the plot. There are two FOI outliers each at FOI = 1.2 for the S+G and G only households that have been removed (herd sizes 22 and 38 sheep and goats).

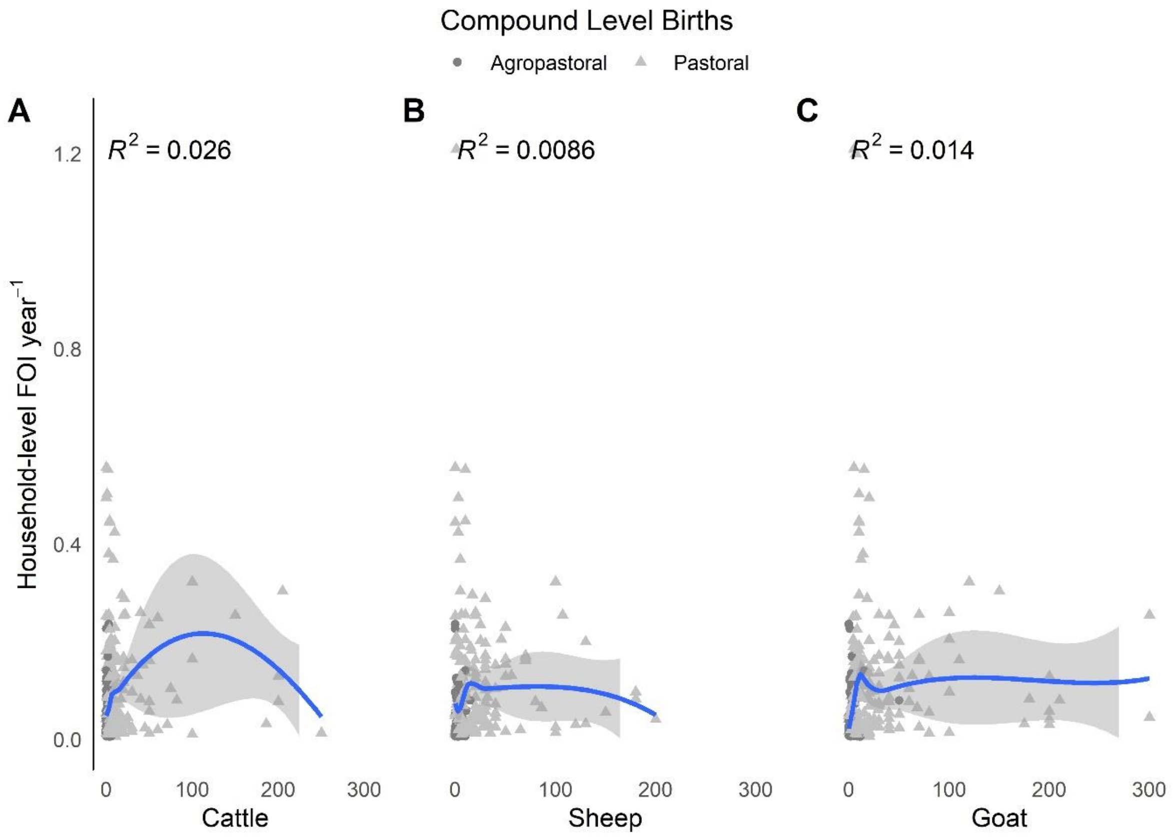

Figure 8. Household herd FOI had a very weak relationship with compound-level birth counts in the past 12 months. FOI appears to grow most rapidly when births were between 1 and 15 animals after which the relationship leveled off. Here, data is stratified by species and production system type. Locally estimated scatterplot smoothing (LOESS) fit to the data with confidence interval. Birth counts greater than 300 were not plotted (3 for sheep, 2 for goats) but are included in the calculation.

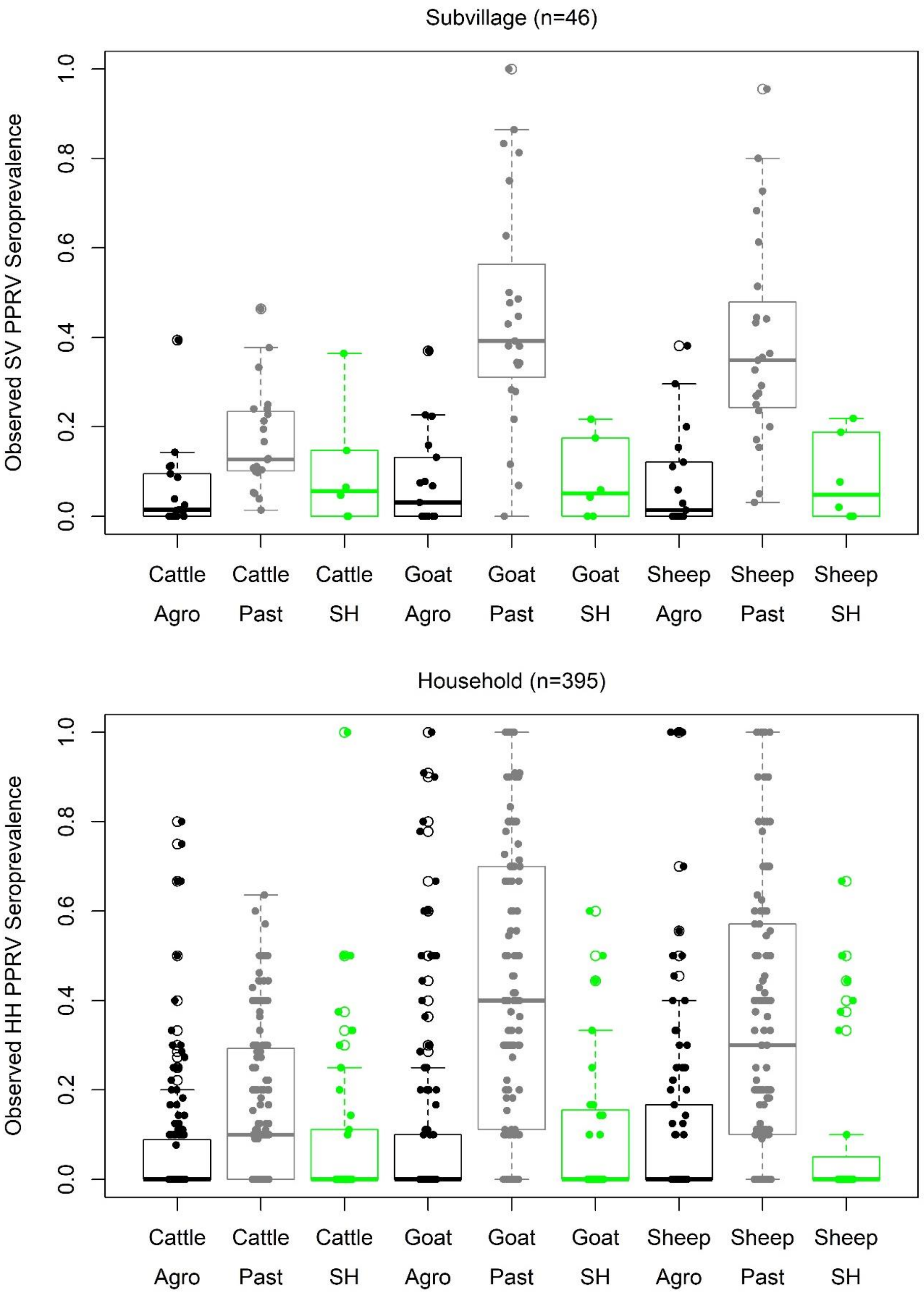

Figure 9. Boxplots of variation in PPRV seroprevalence by species and production system at the sub-village level and household herd level. Jittered dots are seroprevalence proportion estimates of sampled villages, sub-villages, and household herds, respectively. Agro = Agropastoral (Black), Past = Pastoral (Gray), SH = Smallholder (Green). Classifications based on de Glanville et al 2020 [21].

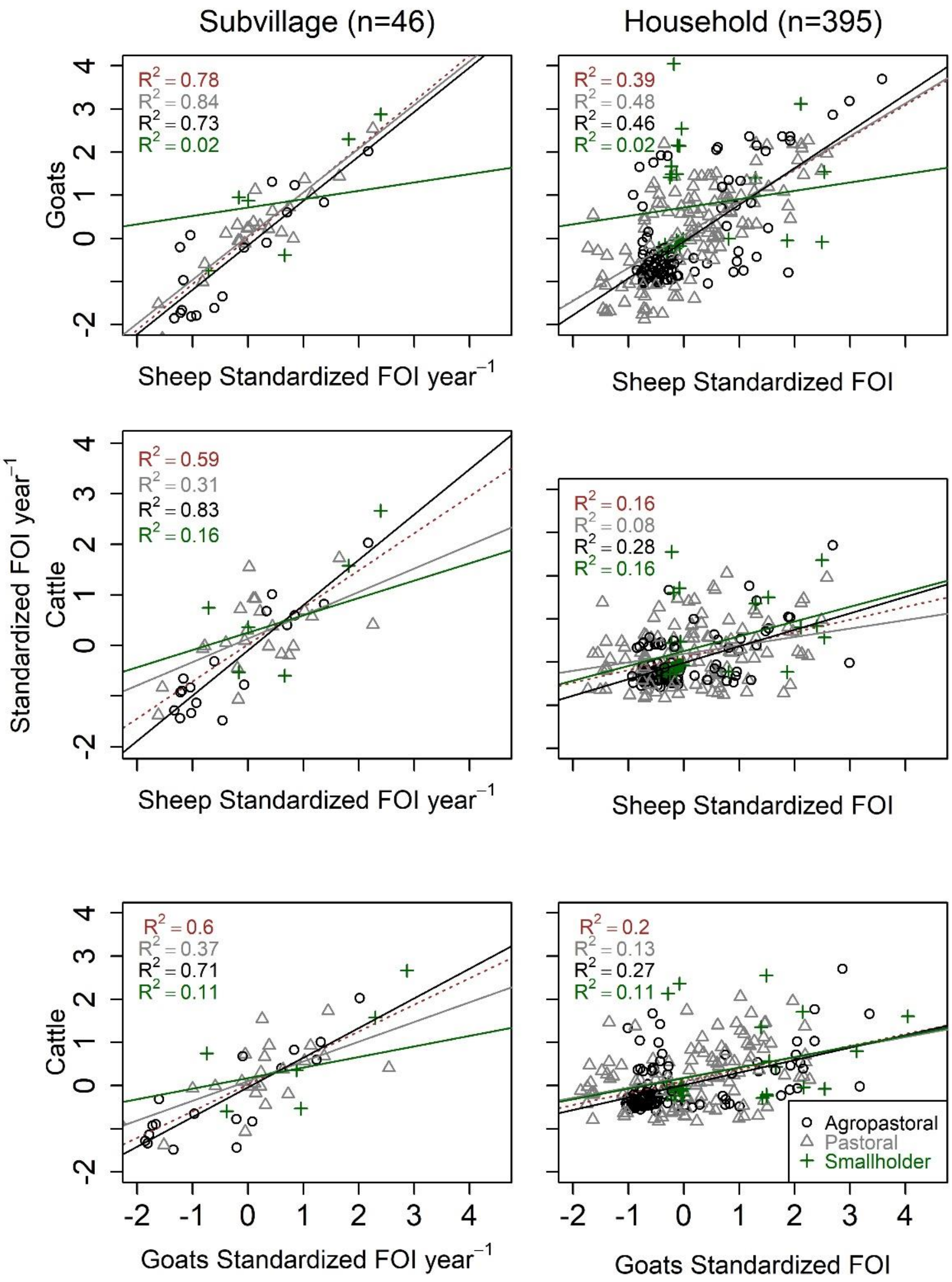

Figure 10. Standardized force of infection (FOI, year^{-1}) estimates by cluster (pastoral, agropastoral, smallholder based on de Glanville et al 2020 [21] classifications) at the sub-village and household herd level by species. Mean FOI is standardized to zero and is in the middle of both axes. Where the FOI was higher or lower for one species, it was also higher or lower than the mean for the second species plotted. These patterns, at all scales, are consistent with what would be expected for cross-species transmission or if a common, external factor was affecting all three species. All correlations were are significant (most $p < 0.001$; all $p < 0.002$). Linear models were fit to each production type and resulting adjusted R^2 is reported for each production type and overall (brown).

Table 1. Sample population characteristics.

	**n (%)**	**PPRV Seropositive n (%, *95% CI*)**
Total	7,115	1,483 (20.8%, *20.0-21.8%*)
Species		
Sheep	1,975 (27.8%)	512 (25.9%, *24.0-27.9%*)
Goats	2,302 (32.4%)	647 (28.1%, *26.3-30.0%*)
Cattle	2,838 (39.9%)	324 (11.4%, *10.3-12.6%*)
Sex		
Female	5,227 (73.5%)	1,303 (24.9%, *23.8-26.1%*)
Male	1,888 (26.5%)	180 (9.5%, *8.2-10.9%*)
Management system		
Agropastoral (AP)	2,775 (39.0%)	155 (5.6%, *4.8-6.5%*)
Pastoral (P)	4,340 (61.0%)	1,328 (30.6%, *29.2-32.0%*)
Age Group†		
Temporary teeth	1,788 (23.9%)	104 (5.8%, *4.8-7.0%*)
1 pair	722 (9.6%)	66 (9.1%, *7.1-11.5%*)
2 pair	635 (8.5%)	86 (13.5%, *11.0-16.5%*)
3 pair	461 (6.1%)	95 (20.6%, *17.0-24.6%*)
4 pair	3,112 (41.5%)	1,013 (32.5%, *30.9-34.2%*)
Full mouth + worn	397 (5.3%)	119 (30.0%, *25.5-34.7%*)
Breed		
Indigenous breed	6,755 (95.0%)	1,446 (21.6%, *20.4-22.4%*)
Cross breed	357 (5.0%)	37 (10.4%, *7.4-14.0%*)
Exotic breed	3 (0.04%)	0 (0%)
Village‡		
Arri (AP)	116 (4.0%)	2 (1.7%, *0.2-6.1%*)
Ilkerin (AP)	317 (10.9%)	44 (13.9%, *10.3-18.2%*)
Kansay (AP)	458 (15.8%)	6 (1.3%, 0.5-2.8%)
Kisimiri (AP)	228 (7.9%)	47 (20.6%, *15.6-26.5%*)
Long (AP)	258 (8.9%)	2 (0.8%, *0.1-2.8%*)
Lositete (AP)	236 (8.1%)	31 (13.1%, *9.1-18.1%*)
Maheri (AP)	480 (16.6%)	1 (0.2%, *0.00005-1.2%*)
Nambala (AP)	322 (11.1%)	10 (3.1%, *1.5-5.6%*)
Sarame (AP)	360 (12.4%)	12 (3.3%, *1.7-5.8%*)
Endanyawish (P)	213 (4.6%)	7 (3.3%, *1.3-6.7%*)
Engikaret (P)	529 (11.5%)	151 (28.5%, *24.7-32.6%*)
Engusero Sambu (P)	328 (7.1%)	50 (15.2%, *11.5-19.6%*)
Kimokowa (P)	329 (7.2%)	117 (35.6%, *30.1-39.1%*)
Komolo (P)	368 (8.0%)	89 (24.2%, *19.9-28.9%*)
Mnjingu (P)	333 (7.2%)	76 (22.8%, *18.4-27.7%*)
Naiti (P)	584 (12.7%)	271 (46.4%, *42.3-50.5%*)
Ngage (P)	356 (7.2%)	152 (42.7%, *37.5-48.0%*)
Oloipiri (P)	371 (8.1%)	121 (32.6%, *27.9-37.6%*)
Ruvu Remitii (P)	496 (10.8%)	230 (46.4%, *41.9-50.9%*)
Sukuro (P)	433 (9.4%)	64 (14.8%, *11.6-18.5%*)

† Age groups by dentition correspond to approximately the following ages in cattle: temporary teeth: 1 month to 1.5 years; 1 pair: 1.5-2.5 years; 2 pair: 2.5-3.5 years, 3 pair: 3.5-4.5 years, 4 pair: 4.5-7 years; full + worn: above 7 years. For sheep and goats: temporary teeth: < 1 year; 1 pair: 1-1.5 years; 2 pair: 1.5-2 years, 3 pair: 2-3 years, 4 pair: 3-5 years; full + worn: above 5 years. ‡Denominators for the villages were the n of the corresponding management system type. CI : Confidence Interval.

Table 2. Demographic characteristics of the 395 surveyed heads of household*.

	**Pastoral (n=203)**	**Agropastoral (n=192)**	**Overall (n=395)**
**Sex**			
Female	10 (4.9%)	18 (9.4%)	28 (7.1%)
Male	193 (95.1%)	174 (90.6%)	367 (92.9%)
**Tribe**			
Arusha	31 (15.3%)	48 (25%)	79 (20.0%)
Barabaig	8 (3.9%)	0 (0%)	8 (2.0%)
Iraqw	3 (1.5%)	91 (47.4%)	94 (23.8%)
Maasai	159 (78.3%)	2 (1%)	161 (40.8%)
Tatoga	2 (0.1%)	0 (0%)	2 (0.5%)
Burunge	0 (0%)	1 (0.5%)	1 (0.3%)
Hehe	0 (0%)	1 (0.5%)	1 (0.3%)
Meru	0 (0%)	32(16.7%)	32 (8.1%)
Luguru	1 (0.5%)	0 (0%)	1 (0.3%)
Nyaturu	0 (0%)	9 (4.7%)	9 (2.3%)
Nyiramba	0 (0%)	2 (1.0%)	2 (0.5%)
Sandawe	0 (0%)	3 (1.6%)	3 (0.8%)
**Education Level**			
No formal	116 (57.1%)	41 (21.4%)	157 (39.7%)
Some primary	22 (10.8%)	24 (12.5%)	46 (8.1%)
Complete primary	55 (27.1%)	116 (60.4%)	171 (43.3%)
Some secondary	4 (2.0%)	1 (0.5%)	5 (1.3%)
Complete secondary	6 (3.0%)	7 (3.6%)	13 (3.3%)
Post secondary	0 (0%)	1 (0.5%)	1 (0.3%)
Some university	0 (0%)	1 (0.5%)	1 (0.3%)
Complete university	0 (0%)	1 (0.5%)	1 (0.3%)
**Grows Crops**			
No	45 (22.2%)	7 (3.6%)	52 (13.2%)
Yes	158 (77.8%)	185 (96.4%)	343 (86.8%)

*138 responses from respondents who were not the head of household but reported on the characteristics of the head of the household.

Table 3. Household-level domestic species composition by production type.

	Pastoral (n=203)	Agropastoral (n=192)	Overall (n=395)
**Cattle**			
Mean (SD)	96.3 (161)	9.07 (8.03)	54.2 (124)
Median [Min, Max]	37.5 [0.00, 1200]	7.00 [1.00, 60.0]	12.0 [0.00, 1200]
Missing	7 (3.4%)	9 (4.7%)	16 (4.1%)
**Goats**			
Mean (SD)	105 (152)	12.0 (12.6)	61.8 (121)
Median [Min, Max]	50.0 [1.00, 1000]	8.00 [1.00, 65.0]	20.0 [1.00, 1000]
Missing	16 (7.9%)	32 (16.7%)	48 (12.2%)
**Sheep**			
Mean (SD)	103 (222)	10.3 (10.0)	64.2 (176)
Median [Min, Max]	40.0 [1.00, 1800]	7.00 [1.00, 57.0]	16.0 [1.00, 1800]
Missing	22 (10.8%)	63 (32.8%)	85 (21.5%)
**Chickens**			
Mean (SD)	260 (1550)	66.3 (753)	159 (1210)
Median [Min, Max]	10.0 [0.00, 10000]	8.00 [1.00, 10000]	10.0 [0.00, 10000]
Missing	40 (19.7%)	16 (8.3%)	56 (14.2%)
**Dogs**			
Mean (SD)	3.10 (2.15)	2.21 (1.57)	2.75 (1.98)
Median [Min, Max]	3.00 [1.00, 12.0]	2.00 [1.00, 9.00]	2.00 [1.00, 12.0]
Missing	39 (19.2%)	85 (44.3%)	124 (31.4%)
**Donkeys**			
Mean (SD)	8.99 (15.3)	2.31 (1.39)	7.11 (13.4)
Median [Min, Max]	4.50 [1.00, 100]	2.00 [1.00, 5.00]	4.00 [1.00, 100]
Missing	37 (18.2%)	127 (66.1%)	164 (41.5%)
**Cats**			
Mean (SD)	87.6 (924)	1.67 (1.20)	48.5 (682)
Median [Min, Max]	2.00 [1.00, 10000]	1.00 [0.00, 6.00]	1.00 [0.00, 10000]
Missing	86 (42.4%)	94 (49.0%)	180 (45.6%)
**Pigs**			
Mean (SD)	NA (NA)	2.27 (1.64)	2.27 (1.64)
Median [Min, Max]	NA [NA, NA]	2.00 [1.00, 10.0]	2.00 [1.00, 10.0]
Missing	203 (100%)	141 (73.4%)	344 (87.1%)
**Birds***			
Mean (SD)	NA (NA)	2.00 (NA)	2.00 (NA)
Median [Min, Max]	NA [NA, NA]	2.00 [2.00, 2.00]	2.00 [2.00, 2.00]
Missing	203 (100%)	191 (99.5%)	394 (99.7%)

	**Pastoral (n=203)**	**Agropastoral (n=192)**	**Overall (n=395)**
**Other Animals****			
Mean (SD)	NA (NA)	5.00 (4.24)	5.00 (4.24)
Median [Min, Max]	NA [NA, NA]	5.00 [2.00, 8.00]	5.00 [2.00, 8.00]
Missing	203 (100%)	190 (99.0%)	393 (99.5%)

'Missing' indicates how many households did not report having these domestic species.

* Only one household reported managing birds.

** Other animals category reported only rabbits in two households.

Table 4. Generalized linear model (GLM) and generalized linear mixed model (GLMM) comparison shows sub-village geographic scale best captures spatial variation in *peste des petits ruminants* virus (PPRV) transmission risk*.

Random Effect	Akaike's Information Criterion
No random effect (GLM)	6677.6
Village (GLMM)	5430.9
Sub-village (GLMM)	5352.6
Household (GLMM)	5421.2

*All GLMMs in this comparison have no fixed effects.
